# Pituitary metastasis as the first manifestation of lung carcinoma

**DOI:** 10.1002/ccr3.6601

**Published:** 2022-12-12

**Authors:** Sara Amaral, Alexandra Matias, Bruno Bouça, Inês Manique, Ana Palha, Luísa Cortez, Luís Cerqueira, Dalila Forte, Amets Sagarribay, Eduardo Dutra, Miguel Cristóvão, Carlos Pontinha, Manuela Mafra, José Silva‐Nunes

**Affiliations:** ^1^ Department of Endocrinology, Diabetes and Metabolism Centro Hospitalar Universitário Lisboa Central Lisbon Portugal; ^2^ Nova Medical School/ Faculdade de Ciencias Medicas Universidade Nova de Lisboa Lisbon Portugal; ^3^ Department of Neurorradiology Centro Hospitalar Universitário Lisboa Central, Hospital de Curry Cabral Lisbon Portugal; ^4^ Department of Neurosurgery Centro Hospitalar Universitário Lisboa Central, Hospital de Curry Cabral Lisbon Portugal; ^5^ Department of Pathological Anatomy Centro Hospitalar Universitário Lisboa Central, Hospital de Curry Cabral Lisbon Portugal; ^6^ Health and Technology Research Center (H&TRC), Escola Superior de Tecnologia da Saude de Lisboa Lisbon Portugal

**Keywords:** diabetes insipidus, hypopituitarism, lung carcinoma, pituitary, pituitary metastases

## Abstract

Pituitary metastases are rare. Clinical presentation could range from asymptomatic to panhypopituitarism or local symptoms. We present a case report of a 43‐year‐old male patient with a new onset headache, visual disturbances, and panhypopituitarism. The investigation led to the diagnosis of pituitary metastasis as the first manifestation of underlying lung cancer.

## Abstract 2

With rising incidence of breast and lung malignancy, headache/visual field disturbance or signs of pituitary hormone deficiency might be the presenting feature of pituitary metastatic disease. A rapid growth of a sellar lesion and/or the presence of diabetes insipidus along with a sellar mass should raise the suspicion for malignancy.

## 1 INTRODUCTION

Pituitary metastasis is a rare manifestation of a malignant neoplasm. Its clinical detection may be underestimated since its prevalence may reach 4%, considering autopsy results.^1^ They could be the first manifestation of primary neoplasms in 10% of cases.^2^ Male gender has been described as the most affected, being most frequently diagnosed between 60 and 64 years of age.^2^ After breast, lung is the most common primary tumor metastasizing to the pituitary gland. Twenty‐five percent of all reported cases of pituitary metastasis are of lung primary origin. Small cell lung cancer is the most frequent histological subtype associated with pituitary metastasis.^3^

Clinical manifestations triggered by pituitary metastasis can result from the mass effect on adjacent structures and may depend on the location of the mass in the pituitary gland. Visual field defects, anterior pituitary hormonal deficiency like central hypothyroidism and secondary adrenal insufficiency, and diabetes insipidus have been reported. This last manifestation appears to be less common compared to reports from previous series.^3^ Nevertheless, the development of diabetes insipidus seems to be a determining factor in differentiating the nature of the pituitary lesion. Diabetes insipidus as a first manifestation of pituitary adenoma is rare and in this setting, the clinician should be alerted to a possible malignant nature of the pituitary mass.^4^

The existing treatment options are varied, from surgery, radiotherapy, and chemotherapy. Transsphenoidal surgery has been used to relieve mass effect on the optic apparatus, improving visual complaints.^2^ The prognosis associated with the detection of pituitary metastasis is variable and survival rate ranges from days to several years, depending essentially on the histological subtype of the primary tumor.^5^

## 2 CASE HISTORY

A 43‐year‐old male patient was admitted to the Emergency Department in November 2020 reporting a three week progressively worsening right‐side headache accompanied by photophobia. It was not associated with visual impairment, nausea, anorexia, weight loss, seizures, fever, or weakness and there was no previous history of trauma. He started administering paracetamol 1000 mg bid with no pain relief. He had history of non‐treated dyslipidemia and he was a current smoker with a 20‐pack‐year history of tobacco use. Upon examination he was anxious with normal vital signs (blood pressure 119/70 mmHg, heart rate 93 bpm, respiratory rate 15 rpm, oxygen saturation 97%, and tympanic temperature 36.7°C). Chest auscultation, abdominal examination, and neurological examination were unremarkable. Laboratory evaluation revealed a normal blood count and a normal kidney and liver function. Computed tomography (CT) of the brain was also normal with mild sinuses inflammation. He was sent home on nonsteroidal *anti*‐*inflammatory* medication.

Despite analgesic medication, the pain did not improve. He had a Neurology Consultation two weeks later and performed a brain magnetic resonance imaging (MRI) reporting some i*nflammatory* changes in the paranasal *sinuses* with no other alterations. The patient was reassured with the results of the MRI findings and was medicated with lysine acetylsalicylate with mild improvement.

**FIGURE 1:**
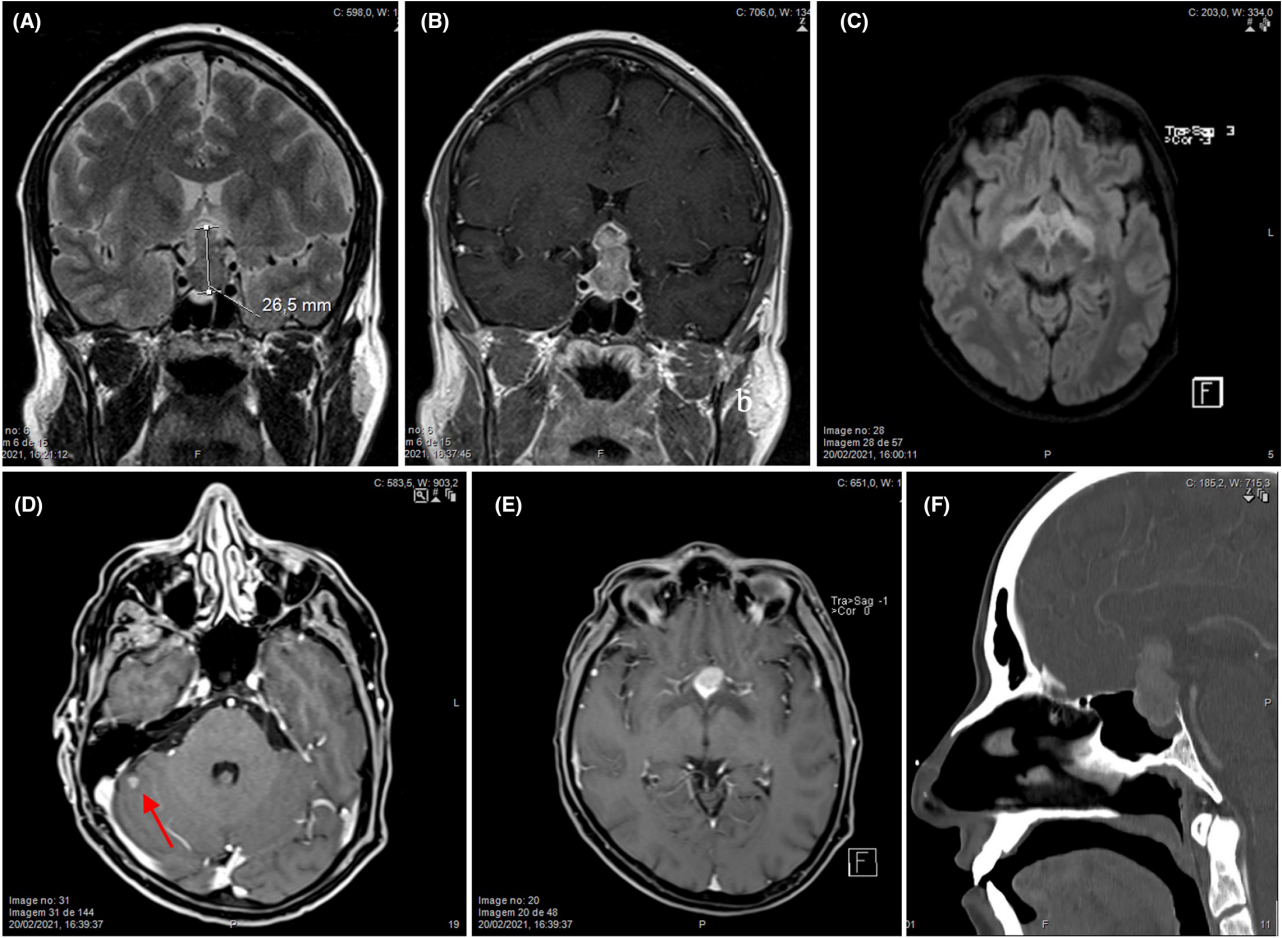
Coronal T2 (A) and T1 post‐gadolinium (B), axial T2 FLAIR reconstructed (C), axial and coronal T1 post‐gadolinium reconstructed (D, E) scans: 27 mm sellar and suprasellar lesion with marked superior shift of the optic chiasm and edema of the optic tracts and a 5 mm nodular enhancing lesion at the gray/white matter interface of the right cerebellar hemisphere (arrow, D). Sagittal CT section with post iodinated contrast medium (F) depicts erosion of sellar floor and dorsum indicating aggressive fast‐growing behavior.

In February 2021, he started complaining about rapidly increasing visual disturbances with significant loss of vision in the left eye and temporal hemifield of the right eye. On confrontation visual fields testing, he had apparent temporal hemianopsia in the right eye. Ophthalmological examination also showed a diffuse reduction in sensitivity to stimuli on left eye with a relative afferent pupillary defect. He had an urgent brain MRI done that revealed a 33.3 mm (anteroposterior) × 44.1 mm (transverse) × 47.3 mm (longitudinal) sellar and suprasellar lesion lobular, isointense, heterogenous with extension along the floor of the 3rd ventricle in bilateral contact with the internal carotid arteries, with no signs of invasion of the cavernous sinus; additionally, a superior shift of the optic chiasm was observed as well as an unspecific cerebellar lesion with 5 mm (Figure 1).

## 3 INVESTIGATIONS AND TREATMENT

Hormonal evaluation was obtained (Table 1) revealing a secondary hypothyroidism (fT4 0.54 ng/dl (0.70–1.48), TSH 0.86 uUI/ml (0.35–4.94)) and hypogonadotropic hypogonadism (total testosterone 0.030 ng/ml (1.424–9.231), FSH 0.35 mUI/ml (0.95–11.95), LH <0.09 mUI/ml (0.57–12.07)). IGF‐1 and prolactin levels were normal (151 ng/ml (58.2–219) and 20.96 ng/ml (3.46–21.4), respectively). Low random cortisol (2.60 μg/dl) with inappropriately low‐normal ACTH levels (12.70 pg/ml) prompted concern for secondary adrenal insufficiency. The patient started dexamethasone 12 mg/day due to cerebral edema and levothyroxine 50 μg/day.

**TABLE 1:** Hormonal evaluation following the diagnosis of sellar and suprasellar lesion

Cortisol	2.60 μg/dl	
ACTH	12.70 pg/ml	(ND‐46 pg/ml)
TSH	0.86 μUI/ml	(0.35–4.94)
Free T4	0.54 ng/dl	(0.70–1.48)
Total testosterone	0.030 ng/ml	(1.424–9.231)
LH	<0.09 mUI/ml	(0.57–12.07)
FSH	0.35 mUI/ml	(0.95–11.95)
IGF‐1	151 ng/ml	(58.2–219)
Prolactin	20.96 ng/ml	(3.46–19.40)

Before admission to Neurosurgery Ward, the patient had a screening positive polymerase chain reaction (*PCR*) *test* for *COVID*‐19 and he was then admitted to a specific COVID‐19 ward. He remained without symptoms of SARS‐CoV‐2 infection. On Day 5, he started complaining about polydipsia and polyuria (urine output 5100 ml/24 h) and presented with mild hypernatremia 147 mEq/L.^6^ A diagnosis of diabetes insipidus was suspected and therapeutic test with desmopressin was performed with clinical improvement. On Day 10, a new PCR test for SARS‐CoV‐2 infection was negative and he was transferred to the Neurosurgery Department.

Given the high suspicion for malignancy, the patient also had a thoraco‐abdominal‐pelvic CT done; it revealed atelectasis and reduction in the caliber of the bronchus on the left lower lobe of the lung with a small left basal pleural effusion, heterogeneous tissue densification with involvement of the left branch of the pulmonary artery and the segments to the lower lobe and small non‐measurable infracarinal and right pulmonary hilum ganglia.

**FIGURE 2:**
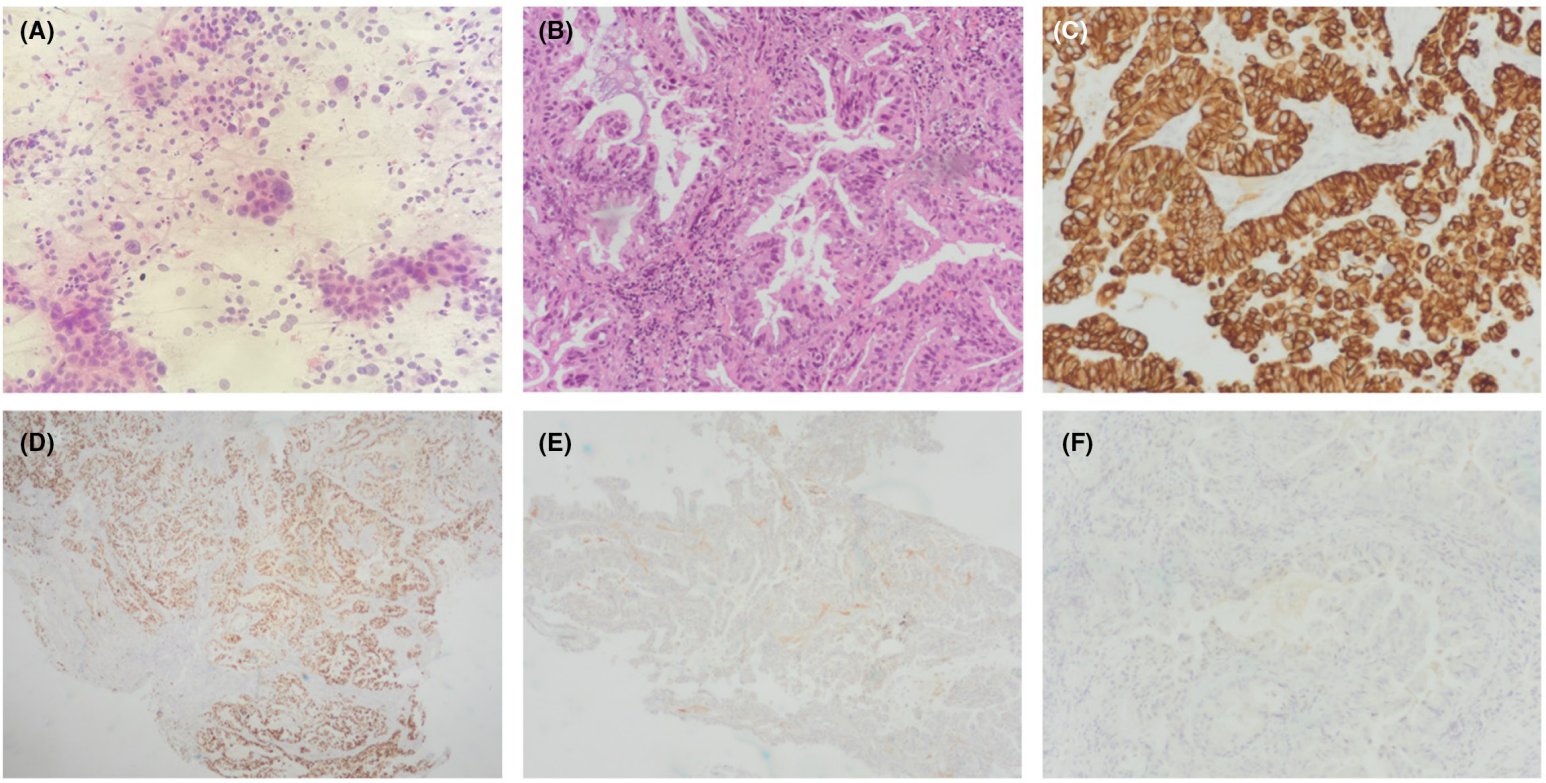
(A) Intraoperative consultation—squash smear of sellar lesion showing aggregates of large and pleomorphic cells; (B) Hematoxylin‐eosin stain showing glandular malignant neoplasia, with mucin production; (C) immunohistochemistry showing positivity for CK7; (D) positivity for TTF1; (E) positivity for Napsin A; (F) negativity for CK20.

He underwent transsphenoidal endonasal endoscopic resection of the pituitary mass in March 2021. A highly‐vascularized, capsulated sellar and suprasellar lesion was encountered and dissected from the frontal lobe, optic chiasm and the anterior communicating artery complex. The tumor was also adherent to the floor of the third ventricle. A gross‐total removal was obtained. Intraoperative histological examination revealed large, pleomorphic cells, with irregular nuclear contours and chromatin, sometimes forming aggregates. Histopathology in the definite paraffin examination confirmed the suspicion of a pituitary metastasis of primary lung carcinoma. It turned out to be an adenocarcinoma, of papillary pattern, characterized by positive immunostaining for CK7, TTF1, Napsin A and negative for CK20 (Figure 2). The final pathological diagnosis was rendered as metastatic carcinoma of lung origin.

**FIGURE 3:**
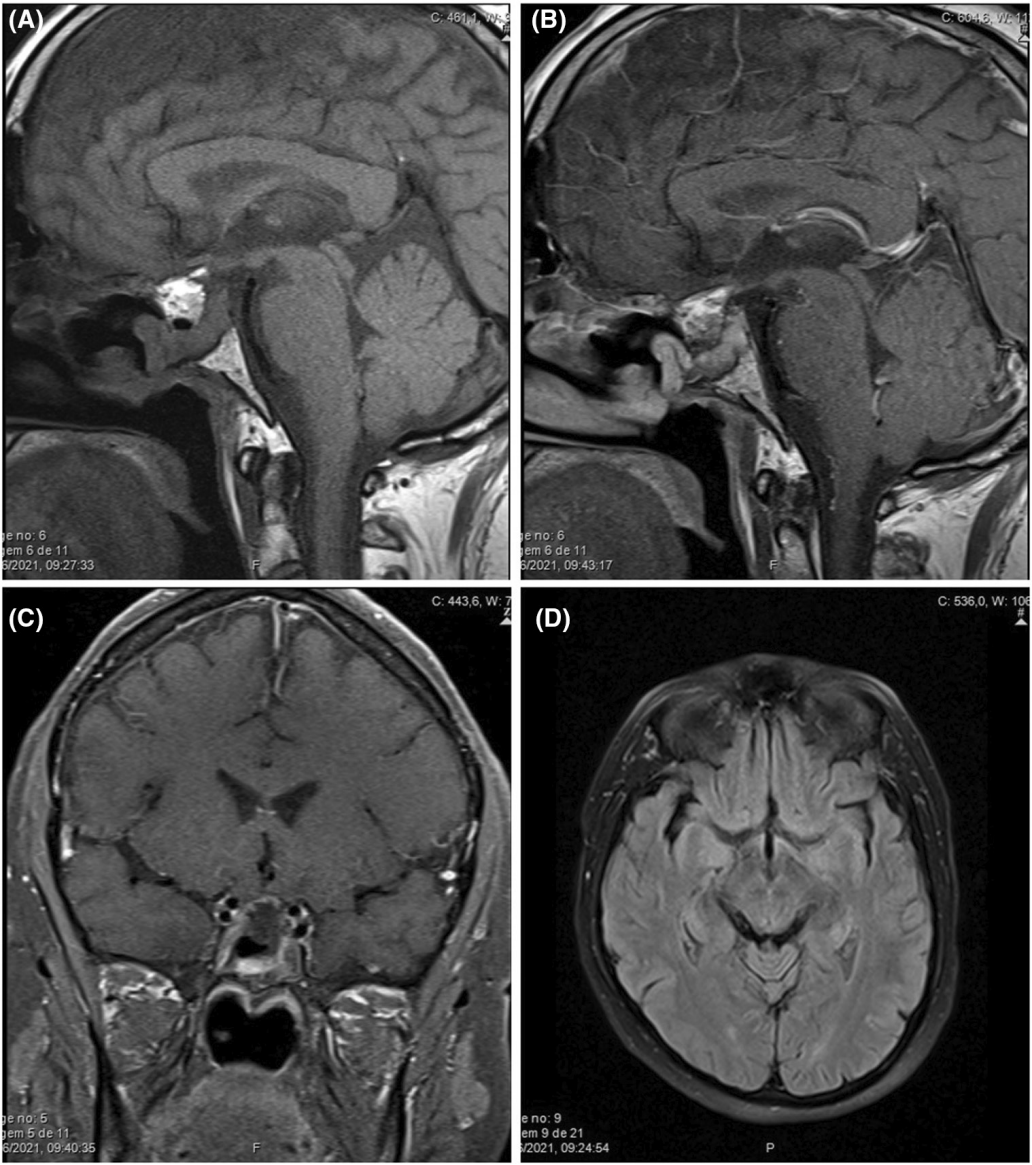
Post‐operative MR sagittal T1 (A) and T1 post‐gadolinium (B), coronal T1 fat suppressed post‐gadolinium (C), axial T2 FLAIR (D) scans: complete removal of the sellar and suprasellar lesion with fat filling of the surgical space (T1 hyperintense, hypointense in the T1 fat suppressed sequence), and complete resolution of the optic pathway edema (T2 FLAIR).

After surgery, the patient reported improvement of visual disturbances along with significant clinical improvement. Post‐surgery MRI showed apparently complete resection of the sellar and supra‐sellar lesion with no signs of residual tumor (Figure 3).

Neuro‐ophthalmological assessment showed a bilateral compressive/infiltrative optical neuropathy.

## 4 OUTCOME AND FOLLOW‐UP

A multidisciplinary approach was made with Endocrinology, Neurosurgery, Oncology, and Radiotherapy. The patient received a 15 Gy single‐fraction stereotactic radiosurgery in the cerebellar lesion and 25 Gy in 5 fractions in sellar region. NGS lung cancer panel was negative for mutations in ALK, ATK1, BRAF, CDK4, CTNNB1, DDR2, EGFR, FGFR2, FGFR3, HER2, HER3, HER4, JAK2, JAK3, KRAS, MAP2K1, MET, NRAS, PIK3CA, ROS1, and rearrangements in ALK, BRAF, FGFR3, NTRK, RET, and ROS1. Two months after surgery, fluorodeoxyglucose (FDG)‐positron emission tomography (PET) showed a focal area of FDG uptake in left lung and multiple musculocutaneous metastasis. Thus, in June 2021, the patient was started on adjuvant chemotherapy with cisplatin and pemetrexed; additionally, he was submitted to radiotherapy on the right inguinal lesion. Two months later, a significant size reduction of the lung lesion was observed, and a total resolution of musculocutaneous lesions was confirmed by FDG‐PET.

However, in September 2021, chemotherapy was stopped due to acute kidney injury that was attributed to cisplatin and pemetrexed. Radiological monitoring showed stable disease until January 2022. Then, he started complaining about progressively need of larger doses of desmopressin to control urine output. Pituitary MRI showed tumor local recurrence and TAP‐CT showed growth of the lung lesion. He started second‐line therapy with nivolumab in February 2022. In the last Endocrinology appointment, he was also medicated with hydrocortisone 20 mg/day, levothyroxine 50 μg/day, desmopressin sublingual 0.30 mg/day, and testosterone i.m. 250 mg per month.

## 5 DISCUSSION

Pituitary metastases are rare and account for less than 1% of surgeries to the pituitary gland. Although neoplasms from almost every tissue have been reported to metastasize to the pituitary, breast, and lung cancer account for approximately two‐thirds of pituitary metastasis; breast cancer is the most common cause for pituitary metastasis among women and lung cancer among men.^7^ One possible explanation could be the fact that these are the two most common tumors in female and male, respectively, and they are the most common cause of brain metastasis.^8^ In fewer than 10% of cases, pituitary metastasis can be the first manifestation of an occult malignancy.^9^ Around 84% of pituitary metastasis occur in the pituitary posterior lobe.^10^ This predominant involvement of the posterior lobe may be explained by the lack of direct arterial blood supply of the anterior lobe. The posterior lobe is supplied by the hypophyseal arteries, whereas the anterior is perfused indirectly via a portal venous system descending from the hypothalamus.^9^ Another explanation may be the fact that posterior lobe has a wider area of contact with the sella turcica and the adjacent dura, and metastasis can spread contiguously via this route. The metastatic deposits in the anterior lobe are usually the result of contiguous invasion from the posterior lobe.^8^

Due to predilection for metastasis to the posterior lobe, the most common hormonal abnormality is diabetes insipidus with an estimated incidence of 70%.^4^ However, pituitary metastasis can also be diagnosed incidentally in asymptomatic patients by radiological evaluation. In a majority of cases, these patients are known to have malignant disease.^10^ Our case report is remarkable for the unusual initial presentation of a lung cancer. The first symptoms were headache that later was associated with visual disturbances. In this clinical report, diabetes insipidus appeared a few days later after the diagnosis of panhypopituitarism and the initiation of hormone replacement. Central diabetes insipidus is known to be masked by adrenal insufficiency and uncovered by subsequent steroid therapy. The glucocorticoid deficiency impairs free water excretion via both arginine vasopressin (AVP)‐dependent and independent mechanisms. In case of glucocorticoids deficiency, the effects of AVP are amplified. Cortisol induces relative resistance of the V2 receptor to AVP, which decreases the translocation of type 2 aquaporins for water reabsorption. Hypocortisolism also results in renal sodium loss and volume depletion, which are potent stimulators for appropriately increasing the release of AVP. Another mechanism is related to corticotrophin‐releasing hormone (CRH) neurons in the paraventricular nucleus that stimulate the release of ACTH and AVP. CRH is upregulated in states of glucocorticoids deficiency and, thus, causes release of AVP. Lastly, glucocorticoid deficiency also leads to a decrease in the stroke volume and cardiac output, resulting in non‐osmotic stimulation of AVP secretion.^11^ Hence, it is important to predict DI in such patients when they are treated with glucocorticoids for adrenal insufficiency.

**FIGURE 4:**
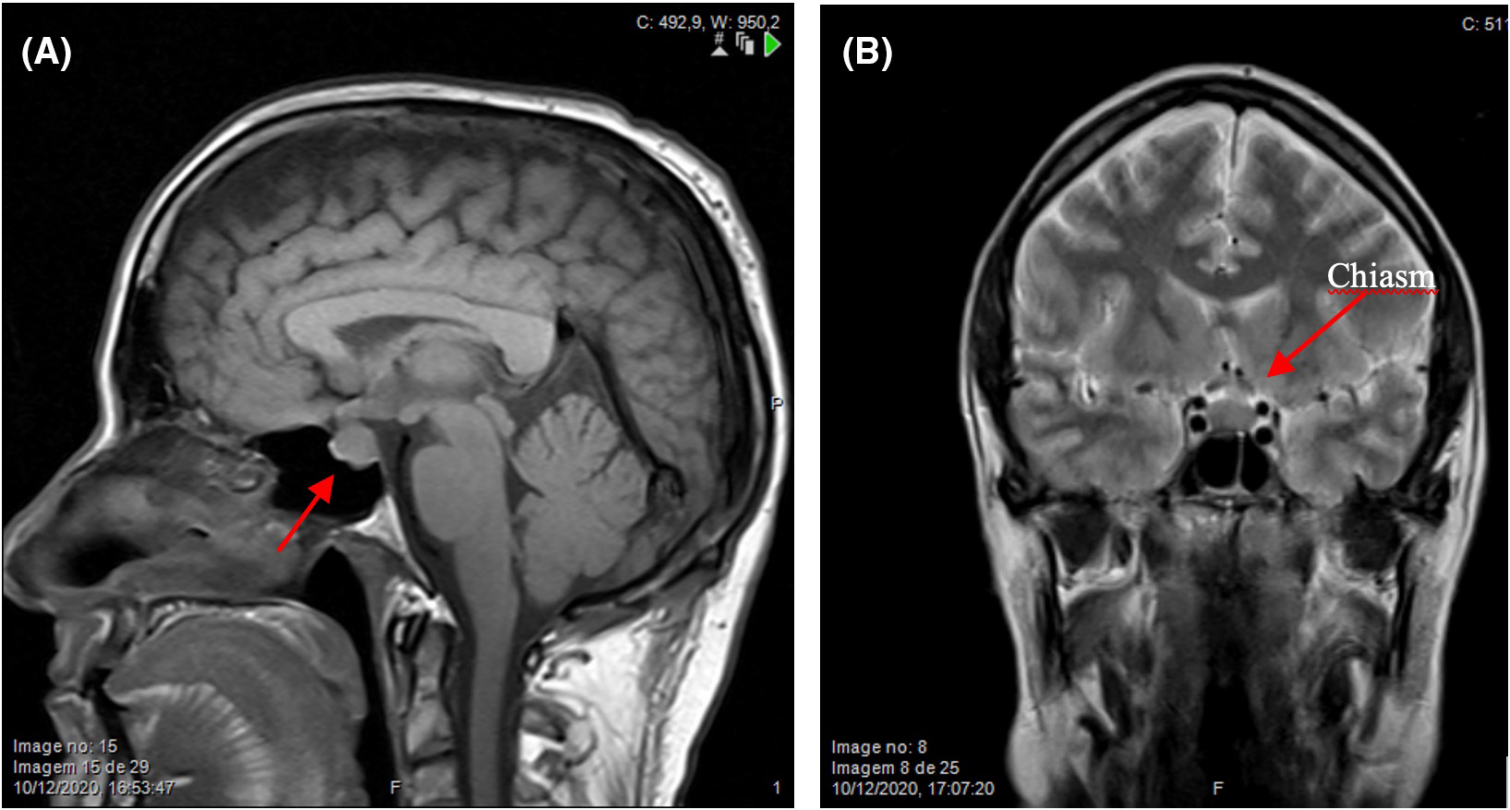
Head MR scan sagittal T1 (A) and coronal T2 (B) sections: 10 mm sellar e suprasellar lesion with slight superior shift of the optic chiasm.

The gold standard for radiological evaluation of a sellar lesion is MRI. The first performed MRI did not describe any lesion. After the diagnosis, the images of the first MRI were reviewed by the Pituitary Team with the identification of a 10 mm sellar e suprasellar lesion with a slight superior shift of the optic chiasm (Figure 4). This abnormality was not reported in the first MRI, probably because this was a brain MRI with thicker cuts and not specifically targeting the pituitary region.

In this report, the rapid growth 2 months apart and the finding of a second lesion raised the suspicion for malignancy. Some other features that suggest pituitary metastasis are thickened pituitary stalk, invasion of the cavernous sinus, bone erosion and invasion of surrounding compartments, heterogeneous or ring contrast enhancement and loss of the normal hyperintense signal of the posterior pituitary lobe on T1 weighted sequences,^9^ and the finding of other tumoral locations. Nevertheless, most of these features are not specific and require differential diagnosis with “aggressive” fast growing PitNET, pituitary carcinoma, pituitary apoplexy, and hypophysitis. In this patient, the added effect of fast progression of optic chiasm compression and visual pathway edema explain the visual disturbances. The fast growing of a sellar/suprasellar lesion, more than doubling its volume in 2 months, the exuberant optic pathway edema suggesting infiltration, erosion of the sellar floor and dorsum and the finding of a second lesion in the gray/white matter interface of the right cerebellar hemisphere strongly indicated metastatic disease, with pituitary carcinoma as an alternative diagnosis.

Treatment is usually palliative and depends on the symptoms and the extension of the systemic disease.^8^ Surgical exploration and decompression are essential for obtaining and histological diagnosis or if suprasellar extension causes severe pain or progressive visual deterioration. Endoscopic transsphenoidal excision and debulking do not affect survival rate but have shown to improve the quality of life by relieving symptoms such as headache and visual disturbances like in this patient. Local radiotherapy and/or chemotherapy (especially if widespread disease is present) are recommended as the adjuvant treatment in combination with hormonal replacement.^10^ Prognosis is usually poor due to the aggressiveness of the primary neoplasia. The mean survival rate in different clinical series varies between 7 and 17 months.^4^

## AUTHOR CONTRIBUTIONS

SA, AM, BB, IM, AP, and LC provided or directed ward care, investigation, and management for the patient. SA wrote the manuscript. LC provided the CT and MRI findings and images. DF and AS performed the surgery. ED, MC, CP, and MM provided the histological images. JSN reviewed the final draft of the case.

## CONFLICT OF INTEREST

The authors declare that the research was conducted in the absence of any commercial or financial relationships that could be construed as a potential conflict of interest.

## CONSENT

Written informed consent was obtained from the patient for the publication of this article.

## Data Availability

Data sharing not applicable to this article as no datasets were generated or analysed during the current study.

## References

[1] Kominos J , Vlassopoulou V , Protopapa D , et al. Tumors metastatic to the pituitary gland: case report and literature review. J Clin Endocrinol Metab. 2004;89(2):574‐580. 14764764 10.1210/jc.2003-030395

[2] Alhashem A , Taha M , Almomen A . Pituitary metastasis of lung adenocarcinoma: case report and literature review. Int Journ Surg Case Rep. 2020;67:98‐101. 10.1016/j.ijscr.2020.01.013 PMC7016037 32058309

[3] Goulart C , Upadhyay S , Filho L , et al. Newly diagnosed Sellar tumors in patients with cancer: a diagnostic challenge and management dilemma. World Neurosurg. 2017;106:254‐265. 28673886 10.1016/j.wneu.2017.06.139

[4] Quintero B , Doe K , Bunker B , Chow W , Yavuz S . Pituitary metastasis of small cell lung cancer: two case reports. J Clin Transl Endocrinol Case Rep. 2021;19:100080.

[5] Teears R , Silverman E . Clinicopathologic review of 88 cases of carcinoma metastatic to the putuitary gland. Cancer. 1975;36:216‐220. 1203849 10.1002/1097-0142(197507)36:1<216::aid-cncr2820360123>3.0.co;2-e

[6] Sioutos P , Yen V , Arbit E . Pituitary gland metastases. Ann Surg Oncol. 1996;3:94‐99. 8770309 10.1007/BF02409058

[7] Heshmati H , Scheithauer W , Young J . Metastases to the pituitary gland. Endocrinologist. 2002;12:45‐49.

[8] Assante R , Zampella E , Emanuele N . Incremental value of sestamibi SPECT/CT over dual‐phase planar scintigraphy in patients with primary hyperparathyroidism and inconclusive ultrasound. Front Med. 2019;6:216‐220. 10.3389/fmed.2019.00164 PMC6646520 31380379

[9] He W , Chen F , Dalm B , Kirby P , Greenlee J . Metastatic involvement of the pituitary gland: a systematic review with pooled individual patient data analysis. Pituitary. 2015;18:159‐168. 24445565 10.1007/s11102-014-0552-2

[10] Pinet C , Raholimina V , Ferri R , Kleisbauer J . Panhypopituitarism secondary to pituitary metastases. Presse Med. 2000;29:17‐18. 10682047

[11] Yang LY , Lin S , Xie QB , Yin G . Central diabetes insipidus unveiled by glucocorticoid therapy in a patient with an empty Sella: a case report and literature review. Medicine (Baltimore). 2020;99(43):e22939. 33120853 10.1097/MD.0000000000022939 PMC7581106

